# Review of CRISPR/Cas Systems on Detection of Nucleotide Sequences

**DOI:** 10.3390/foods12030477

**Published:** 2023-01-19

**Authors:** Mengyu Wang, Haoqian Wang, Kai Li, Xiaoman Li, Xujing Wang, Zhixing Wang

**Affiliations:** 1 Key Laboratory on Safety Assessment (Molecular) of Agri-GMO, Ministry of Agriculture and Rural Affairs, Biotechnology Research Institute, Chinese Academy of Agricultural Sciences, Beijing 100081, China; 2 Development Center for Science and Technology, Ministry of Agriculture and Rural Affairs, Beijing 100176, China; 3 Institute of Quality Standards and Testing Technology for Agro-Products, Chinese Academy of Agricultural Sciences, Beijing 100081, China

**Keywords:** CRISPR/Cas-based detection, *trans*-cleavage, genetically modified organisms detection, gene-edited products detection, single-nucleotide polymorphisms detection, challenges and prospects

## Abstract

Nowadays, with the rapid development of biotechnology, the CRISPR/Cas technology in particular has produced many new traits and products. Therefore, rapid and high-resolution detection methods for biotechnology products are urgently needed, which is extremely important for safety regulation. Recently, in addition to being gene editing tools, CRISPR/Cas systems have also been used in detection of various targets. CRISPR/Cas systems can be successfully used to detect nucleic acids, proteins, metal ions and others in combination with a variety of technologies, with great application prospects in the future. However, there are still some challenges need to be addressed. In this review, we will list some detection methods of genetically modified (GM) crops, gene-edited crops and single-nucleotide polymorphisms (SNPs) based on CRISPR/Cas systems, hoping to bring some inspiration or ideas to readers.

## 1. Introduction

Transgenic technology has been applied in plants, animals, microorganisms and other fields. According to the data of International Service for the Acquisition of Agri-biotech Applications (ISAAA) in 2019, biotech crops were planted by 29 countries. China grew 3.2 million hectares of biotech crops (2% of the global total), and this was predicted to increase in due time globally [1]. In many countries and international organizations, relevant legislation, regulatory measures and evaluation criteria have been adopted to guarantee food traceability, safety supervision of genetically modified (GM) crops and freedom of choice for consumers [2,3,4,5]. With large-scale planting an application of global GM crops and frequent trade circulation under global integration, as well as the continuous promotion of the transgenic industrialization process of China, the task of transgenic supervision will become more and more important, thus making the detection of GM organisms (GMOs) particularly essential [6,7]. In order to implement the No.1 Central Document in 2021, better supervision capacity for GMOs and establishment of a simple, fast, accurate and economic transgenic detection method is imperative, and its requirements will become higher and higher. Polymerase chain reaction (PCR) [8], multiplex PCR [9,10,11,12,13], quantitative PCR (qPCR) [14,15,16,17], droplet digital PCR (ddPCR) [18], loop-mediated isothermal amplification (LAMP) [19,20,21], recombinase polymerase amplification (RPA) [22,23], next-generation sequencing (NGS) [24,25,26,27,28,29], Southern blot [30,31,32], gene chip [33,34] depending on the nucleic acid-based methods, and enzyme-linked immunosorbent assay (ELISA) [35,36], lateral flow assay (LFA) [37], Western blot [38,39] depending on immunological methods, and electrochemical [40], surface plasmon resonance (SPR) [41], and piezoelectric genosensors [42] are commonly used to detect GMOs. The detection of GMOs is mainly based on protein and nucleic acid. Protein-based detection methods often require the preparation of antibodies, which have a long cycle, high cost, and can only detect foreign proteins with limited detection targets. Moreover, protein-based detection methods can only detect fresh or primary samples of crops, and have limitations on the detection of processed or deeply processed products. The nucleic acid-based detection methods are more accurate, reliable, stable and widely used. Among many nucleic acid detection methods, PCR technology is one of the most developed transgenic detection methods, with accurate results, high sensitivity and strong specificity. PCR has been used as the standard test method for food regulations in many countries.

In recent years, clustered regularly interspaced short palindromic repeats/CRISPR-associated proteins (CRISPR/Cas) [43] has become the most popular tool to create a new situation for gene function research and biological breeding [44,45]. Gene-edited products are divided into three categories according to the different repair mechanisms after double-stranded DNA (dsDNA) breaks. Site-directed nuclease systems 1 (SDN1) refers to the fact that no template or any exogenous gene is introduced, only one or a few base insertion or deletion (indels) and substitution of nucleotides. SDN2 refers to an introduced homologous template, which leads to one to several base mutations (<20 bp) in the genome through homologous recombination. SDN3 refers to the insertion of large exogenous genes at target sites through homologous recombination. After gene editing of diploid plants, a single cell of the plant will produce three kinds of mutation results–single allele mutation, also known as heterozygous mutation, double allele mutation, in which two alleles have different types of mutations, and homozygous mutation, where two alleles have the same mutation [46]. Gene editing is site-directed modification of the genome, leaving fewer traces in the recipient. Gene editing is different from transgenic technology, which inserts genetic material into the recipient, and the regulatory measures differ from country to country. The current measures taken by our country are that if the gene-edited products contain exogenous genes, they will be regulated as GMOs. If not, they can be simplified. Therefore, the detection strategy for GMOs and gene-edited products with and without exogenous genes is different, especially SDN1 and SDN2. Sanger [47], NGS [48,49,50], T7 endonuclease I (T7EI) [51,52,53] and restriction fragment length polymorphism (RFLP), also known as the cleaved amplified polymorphic sequence (CAPS) [54,55,56], are frequently used in scientific research and can be used to detect gene-edited products. In addition, amplified fragment length polymorphism (AFLP) [57,58], at critical temperature PCR (ACT-PCR) [59,60], the amplification refractory mutation system (ARMS) also known as allele-specific PCR (AS-PCR) [61], ddPCR [57,62], high-resolution fragment analysis (HRFA) [63], high-resolution melting (HRM) [64], heteroduplex mobility assay (HMA) [65,66], single-strand conformational polymorphism (SSCP) [67], polyacrylamide gel electrophoresis (PAGE) [68], and ligation detection reaction (LDR) [69] can be used in detection.

Among the regulatory requirements for GMOs or gene-edited products in China is the on-site inspection results. However, most of the above detection technologies require complex pretreatment of samples, precision instruments, professional steps and analysis, and are time-consuming, so they are not portable for rapid on-site detection. Therefore, new detection methods for biotechnology products are urgently needed. CRISPR/Cas not only plays an important role in gene editing, but also serves as a tool for molecular detection based on *trans*-cleavage activity. CRISPR/Cas has been successfully applied in clinical diagnosis, food safety, biological breeding and others. At present, Class 2 systems, represented by Cas9, Cas12a (Cpf1), Cas12b, Cas13a (C2c2) and Cas14a (Cas12f1), are the most studied and used single-protein effectors, and have the advantages of simple operation, high specificity and sensitivity [70]. For Cas12, Cas13 and Cas14, when the guide RNA captures the nucleic acid targets, the Cas/RNA/target ternary complex forms, activating the *trans*-cleavage activity of Cas to cleavage the single-stranded DNA/RNA (ssDNA/ssRNA) [71,72,73]. The characteristics of Cas9, Cas12, Cas13 and Cas14 are listed in Table 1. When combined with different methods, the CRISPR/Cas system successfully achieved highly sensitive detection of targets. Examples include DNA [74,75,76,77,78,79,80], RNA [81,82,83,84,85,86,87], protein [88,89,90], Na^+^ [91], Pb^2+^ [86], ATP [91,92], uric acid and *p*-hydroxybenzoic acid [93]. In general, CRISPR/Cas systems may be a good choice to achieve ultra-sensitive detection.

In this review, we list some methods for GMOs, gene-edited products and single-nucleotide polymorphisms (SNPs) detection based on the CRISPR/Cas system combined with multiple detection techniques. Then, the current challenges and prospects for targets detection will be discussed in the end, hoping to bring some inspiration or ideas to readers.

## 2. CRISPR/Cas Systems in Detection of GMOs, Gene-Edited Products and SNPs

When crRNA specifically captures the targets, the formation of the Cas/crRNA/target ternary complex will activate the *trans*-cleavage activity of Cas12, Cas13 and Cas14 effectors. Based on the CRISPR/Cas system, it can be used to detect different target nucleotide sequences simply by changing the crRNA. 

Because the detection strategy for GMOs and gene-edited products with and without exogenous genes is different, in this section, we will separately list some CRISPR/Cas-based detection methods for GMOs and gene-edited products. At the same time, there are only a few articles on the detection of gene-edited products. Considering the characteristics of gene editing, the detection methods of mutations of a single base or a few bases can draw from the detection methods of SNPs.

### 2.1. CRISPR/Cas Systems in Detection of GMOs

**Figure 1:**
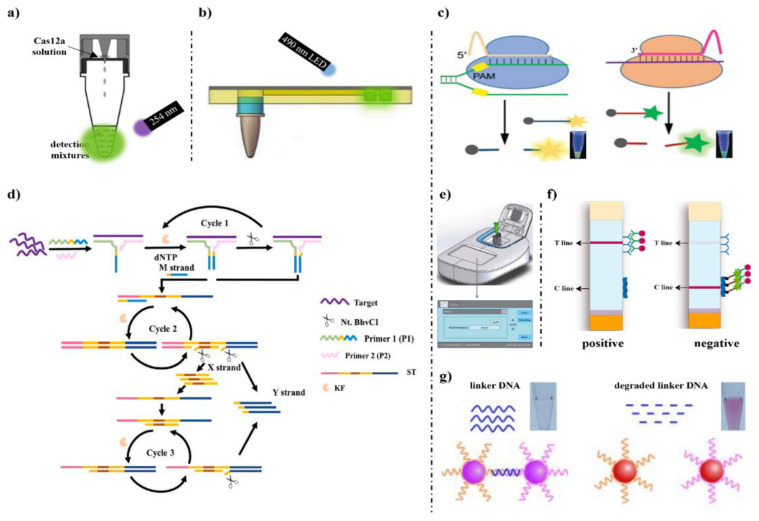
CRISPR/Cas systems in detection of genetically modified organisms (GMOs). (**a**) CRISPR/Cas system for visual detection of *CaMV35S* promoter with a 254 nm UV light [94]. (**b**) Cas12a-PB detection system [95]. (**c**) MPT-Cas12a/13a detection system [96]. (**d**) PE-MC/SDA-CRISPR/Cpf1 detection system [97]. (**e**) Recombinase polymerase amplification (RPA)-Cas12a-FS detection system [98]. (**f**) RPA-Cas12a-LFB detection system [99]. (**g**) A colorimetric gene-sensing platform for detection of transgenic rice [87].

Wu et al. 2020 [94] combined LAMP and CRISPR/Cas12a for visual detection of GM soybean powders with a 254 nm UV light (Figure 1a). This was verified by experiment that the concentration of magnesium ion was important to the CRISPR/Cas12a system. Additionally, the limit of detection (LOD) was 0.05%. The author designed a reaction vessel—after LAMP reaction at the bottom of the tube, the Cas12a reagent at the top of the tube flowed to the bottom of the tube for detection, which was portable and contamination free. In the same year, Wu et al. 2020 [95] developed a portable biosensor for visual dual detection of the *CaMV35S* promoter and *Lectin* gene in soybean powders, which was named Cas12a-PB (Figure 1b). The target DNA were amplified by dual PCR and LAMP in the reaction tube, then the products of amplification were separated into three different chambers, and every chamber contained CRISPR/Cas12a detection systems with an LOD of 0.1%.

Cao et al., 2022 [96] established MPT-Cas12a/13a that combined multiplex PCR and transcription for simultaneous detection of *CaMV35S* and T-*nos* (Figure 1c). Because the CRISPR/Cas12a and CRISPR/Cas13 systems can specifically bind different crRNAs and targets, the systems were used to detect DNA-CaMV35S and RNA-T-*nos*, producing yellow fluorescence at 556 nm and green fluorescence at 520 nm, respectively. The LOD was 13 copies of *CaMV35S* and 11 copies of T-*nos*. Liu et al. 2022 [97] proposed PE-MC/SDA-CRISPR/Cpf1 to detect *CaMV35S* with the LOD down to 14.4 fM (Figure 1d). In the presence of *CaMV35S*, P1 and P2 were designed for hybridization to produce M stand. Then, the M stand can be employed as primers to combine with the strand ST to promote the downstream reaction to produce X and Y stands. The primer X strands can bind to ST, facilitating the next round of reaction and generating a large number of Y strands. The Y strands can activate the *trans*-cleavage of CRISPR/Cpf1, which led to the breakage of the probes. This ingenious amplification method enabled the *CaMV35S* to have a low background interference. Liu et al. 2021 [98] developed a CRISPR/Cas12a-based detection technique by combining RPA, which was named RPA-Cas12a-FS, to detect food-borne microorganisms and GMOs (Figure 1e). Rapid DNA extraction and RPA were used to complete the sample preparation in a short time. After the reaction of CRISPR/Cas12a systems, the samples were measured for fluorescence intensity. The LOD was 10 copies/μL.

Those above methods are based on fluorescence detection, and gold nanoparticle-based colorimetry assay combined with CRISPR/Cas systems is also an attractive detection method. Wang et al., 2020 [99] constructed a highly sensitive procedure based on CRISPR-Cas12a that combined with RPA and LFA, which was named RPA-Cas12a-LFB, for the rapid, visual detection of both P-*CaMV*35S and T-*nos* screening elements (Figure 1f). The test strips were laid with gold nanoparticles labeled FITC antibodies, and the test line (T line) and the control line (C line) were labeled with goat anti-rabbit IgG and biotin ligand, respectively. When there was a target, the dual-labeled reporter (FITC, Biotin) will be degraded, and AuNP complex will gather in the T line for color development, which the result was positive. In contrast, AuNP complex will gather in the C line. The LOD was 10 copies and 0.01% GM crops of Bt11 and MON863 samples. Yuan et al., 2020 [87] had designed a novel colorimetric gene-sensing platform that can visually detect GM rice, African swine fever virus (ASFV), and miRNAs within an hour (Figure 1g). In this method, the *trans*-cleaved substrate was a universal linker ssDNA/ssRNA, which can hybridize to the AuNPs-DNA probes. When there is a target, the linker ssDNA/ssRNA will be cleaved. The probe pair cannot hybridize and thus becomes dispersed. When there is no target, the linker ssDNA/ssRNA will not be cleaved. The probe pair can hybridize to form an aggregated state. Cross-linked and dispersed Au nanoparticle probes will show different colors, and negative and positive samples will be detected. The LOD was 0.01%. The appearance of a test strip enriches the signal output manner.

**Figure 2:**
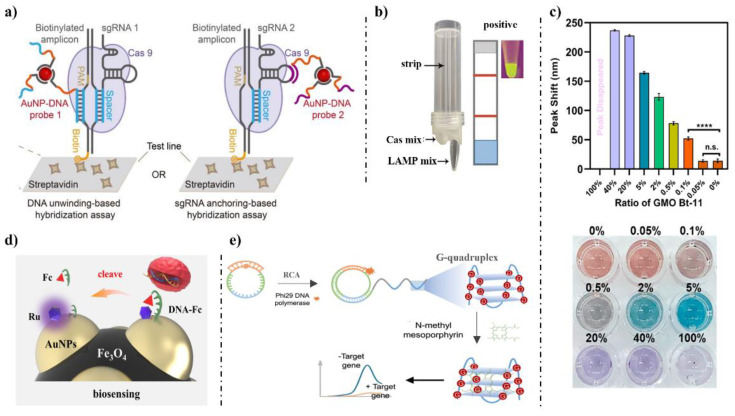
CRISPR/Cas systems in detection of GMOs. (**a**) CASLFA detection system [100]. (**b**) A flow strip or visual detection of P*-CaMV*35S and another three transgenic sequences in GMOs by using a portable device based on CRISPR/Cas [101]. (**c**) A visible detection combined with color change in gold nanorods (GNRs) based on CRISPR-Cas12a [102]. (**d**) A dual-mode electrochemical biosensor for detection of SHZD32-1 without amplification [103]. (**e**) isoCRISPR assay detection system [104].

Wang et al., 2020 [100] combined CRISPR/Cas systems and LFA, which was named CASLFA, to identify *Listeria monocytogenes*, GMOs and ASFV in two strategies (Figure 2a). The AuNP-DNA probes, streptavidin and streptavidin-biotinylated DNA probe were preassembled into the conjugate pad, T line and C line, respectively. Biotin was labeled on the amplicon by PCR or RPA using biotinylated primers. After the samples flow through the conjugate pad, AuNP-DNA probe 1 will hybridize with the target sequences behind the protospacer-adjacent motif (PAM) in the DNA unwinding-based hybridization assay. Or AuNP-DNA probe 2 will hybridize with the target sequences in sgRNA 2 in the sgRNA anchoring-based hybridization assay. The biotin will be captured on the T line, and excess AuNP-DNA probes were captured at the C line. The LOD of the CASLFA method was hundreds of gene copies. Duan et al. 2022 [101] used crude extraction DNA combined LAMP with CRISPR-Cas12a to detect the *pCaMV35S* promoter in transgenic papaya leaves, and another three transgenic sequences in GMOs (Figure 2b). Two rubber chambers were made as reaction chambers for LAMP and Cas solution, and a flow strip was held on the top pf the reaction vessel. After LAMP reaction, the Cas chamber was manually extruded to allow the solution to flow into the LAMP solution. The detection results can be determined by the flow strip or by examining with a 470 nm blue light. Huang et al. 2020 [102] combined CRISPR/Cas systems and recombinase-aided amplification (RAA) with color change in gold nanorods (GNRs) to realize visible detection of NOS terminator in samples (Figure 2c). In the presence of the target, the ssDNA linker was cleaved by Cas12a, and residual magnetic beads (MBs) will be removed by magnet. Sucrose was hydrolyzed by the released invertase, and the produced glucose was oxidized to H_2_O_2_. GNRs were etched by ·OH, and determines the color of the solution. The LOD of this method was 0.1 wt %, and can be semi-quantified of GM ingredients between 0.1 and 40 wt %.

The electrochemical biosensor is also a highly sensitive detection method. Ge et al., 2021 [103] designed a dual-mode electrochemical biosensor for sensitive and reliable detection of GM soybean SHZD32-1 without amplification (Figure 2d). As the signal unit, Fe_3_O_4_@AuNPs/DNA-Fc is Fe_3_O_4_ nanoparticles were coated with AuNPs, on the surface of which ruthenium complex (Ru) and DNA-ferrocene (DNA-Fc) were immobilized. In the presence of the target, the DNA-Fc was cleaved by CRISPR/Cas12a. The electrochemical label Fc will fall off the surface, leading to the decrease in the signal from Fc and the increase signal from Ru. The LOD was 0.3 fM. Zhu et al., 2022 [104] designed an isoCRISPR assay that combined CRISPR/Cas12a systems with rolling circle amplification (RCA) for label-free detection (Figure 2e). When gRNA bound to the target, the RCA primer was degraded and the RCA process ended, leading to a low fluorescence. On the contrary, the primer of RCA can hybridize with the padlock probes that bound with G-quadruplex sequence, thus the amplicon was labeled by G-quadruplex. Then, the RCA amplicon can be detected using N-methyl mesoporphyrin IX (NMM), a G-quadruplex dye, leading to a high fluorescence. The LOD was approximately 45 pM.

### 2.2. CRISPR/Cas Systems in Detection of Gene-Edited Products and SNPs

Gene-edited products, which leave no trace in the recipient, cannot detect universal components in the same way as GMOs. This requires the selection of the specific sequence with a suitable PAM site for detection.

**Figure 3:**
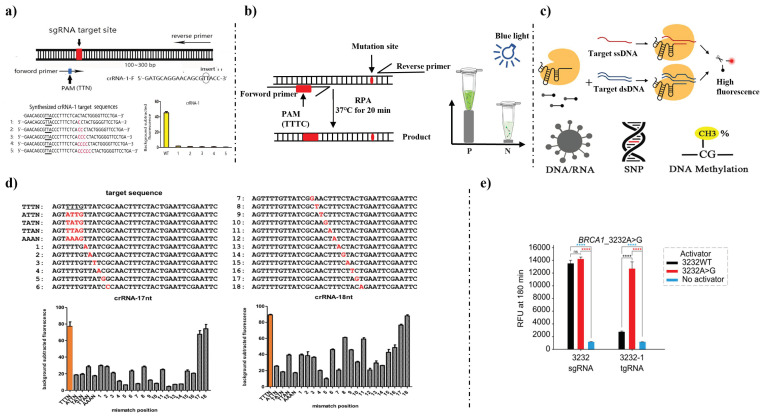
CRISPR/Cas systems in detection of gene-edited products and single-nucleotide polymorphisms (SNPs). (**a**) A biosensing platform for detection of biallelic mutants [106]. (**b**) Cas12aFVD detection system [107]. (**c**) HOLMESv2 detection system [108]. (**d**) HOLMES detection system [109]. (**e**) CDetection detection system [110].

Liang et al., 2018 [105] used preassembled CRISPR/Cas9 and CRISPR/Cpf1 to detect mutations in gene-edited polyploid and diploid plants, which was named PCR/ribonucleoprotein (RNP). This method can distinguish homozygous mutants, biallelic from heterozygous mutations, and also be used for detection of mutagenesis induced by TALEN protein, and mutant screening without affected by background noise SNPs, especially apply to polyploid plants. Furthermore, considering that there might be no suitable PAM sequence near the mutation site, the primers are designed to insert the PAM sequence. Therefore, sequence independent detection was allowed for any site. Xiao et al., 2020 [106] demonstrated that CRISPR/Cas12a systems enabled to identify the biallelic mutants in Thp-1 cells induced by CRISPR/Cas9 and detect different insertions (Figure 3a). Furthermore, this method showed single-base resolution for DNA detection. Wang et al., 2022 [107] developed Cas12aFVD biosensing platform that coupled with RPA for visible detection of mutants in gene-edited rice (Figure 3b). Cas12aFVD can detect single-base mutants with an LOD of 12 copies/μL in 40 min. This method can be applied in the laboratory and on site in one tube.

For gene-edited products of SDN1 and SDN2 with known editing sites and sequences, ACT-PCR, ddPCR, AS-PCR, CRISPR/Cas, etc., one or more methods can be used for preliminary screening. The suspected or positive samples obtained through screening can be further determined by Sanger or NGS, which can greatly reduce the workload. For gene-edited products of SDN3 with known editing sites and sequences, it can be detected according to the current detection strategy of GMOs. For gene-edited products with unknown editing sites and sequences, according to the popular editing sites and common off-target sites, T7EI, RFLP, AS-PCR, HRFA, SSCP, etc., one or more methods can be used for preliminary screening. The suspected or positive samples obtained through screening were further determined by Sanger or NGS. At present, Sanger, NGS, T7EI, and RFLP are widely used, and the application of other methods is relatively few. The selection of detection methods is closely related to gene-editing efficiency, mutation types and plant ploidy. In addition, each method has its own limitations, which can be selected according to specific needs.

Li et al., 2018 [108] created one-step HOLMESv2 with CRISPR-Cas12b to discriminate SNP/single-nucleotide mismatch (SNM) and detect RNA (Figure 3c). When Cas12b combined with asymmetric PCR, Cas12b successfully distinguished the SNP locus without the PAM sequence. That meant it can cleave the ssDNA without a PAM sequence. The author also proved that 18–20 nt sgRNAs were more effective. The LOD of HOLMESv2 was 10^−8^ nM. Li et al., 2018 [109] developed HOMLES to detect SNP loci with a minimum detectable concentration of 10 aM combined (Figure 3d). At the same time, for the PAM mutants and the 1st–7th single-base mismatch, fluorescence signals changed significantly. That meant the detection was more sensitive in this region.

**Figure 4:**
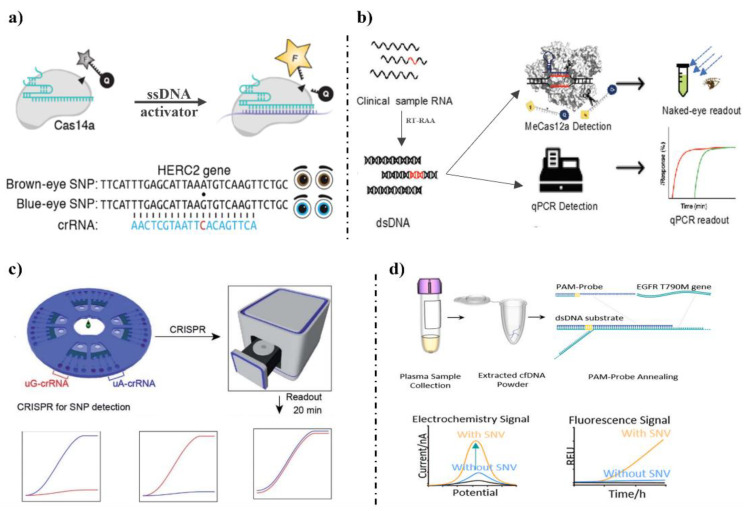
CRISPR/Cas systems in detection of SNPs. (**a**) Cas14a-based detection system of SNP [73]. (**b**) MeCas12a system detection system [111]. (**c**) A CRISPR/Cas system combined with microfluidic technology for automating detection [112]. (**d**) A Cas12a-based detection system with partially double-stranded capture probe to eliminate the need for the PAM sequence [113].

Teng et al., 2019 [110] developed a Cas12b-mediated DNA detection (CDetection) combined with RPA to distinguish the SNP in the human genome using selected tuned guide RNA (tgRNA), achieving single-base resolution detection (Figure 3e). Gootenberg et al., 2017 [71] combined Cas13a with RPA to establish a molecular detection platform, termed SHERLOCK, to distinguish pathogenic bacteria, SNPs of Zika virus (ZIKV) African versus American RNA targets, SNPs, and identify cell-free tumor DNA mutations (Figure 3f). The author chosen five loci of health-related SNPs and benchmarked SHERLOCK detection using 23andMe genotyping data. SHERLOCK distinguished both homozygous and heterozygous genotypes with high significance, and detected SNP-containing alleles as low as 0.1% of background DNA. Additionally, the author found that after lyophilized and subsequently rehydrated, reaction reagents can still be available for detection. Harrington et al., 2018 [73] found that Cas14a required stricter complementarity for recognition of ssDNA, and improved the accuracy detection of SNP without the PAM sequence (Figure 4a). Then, the author used a phosphorothioate-containing primer to amplify HERC2 gene from both blue-eyed and brown-eyed individuals. Cas14a-DETECTR showed strong activation in recognition of the blue-eyed SNP. Ma et al., 2020 [111] described the MeCas12a system to distinguish between SARS-CoV-2 and MERS-CoV and SNPs (Figure 4b). The author tested many divalent ions, and found that manganese ion (Mn^2+^) enhanced the signal of crRNA, effectively improved the Cas12a detection system. The LOD of MeCas12a was five copies of SARS-CoV-2 RNA in 24 patient samples in 45 min.

Microfluidic technology can also be applied in CRISPR/Cas detection systems. Chen et al., 2021 [112] introduced a nucleotide mismatch to improve the universality of the detection of SNP (Figure 4c). The biochip was pre-loaded with CRISPR/Cas12a reagents to automate the process. The biochip can test eight samples at the same time and distinguish the homozygous wild type, the homozygous mutant type and the heterozygous mutant type. Lee et al., 2021 [113] designed a probe containing a PAM sequence and a target capture sequence, and eliminated the need for a PAM sequence with lower noise from the wild type (WT) (Figure 4d). In addition, the author was able to detect up to 10 aM single-nucleotide variants (SNVs) and 0.1% of the mutation with a fluorescence and electrochemical readout. Wang et al., 2022 [114] developed a visualization system based on Cas12a and G4-DNAzyme to identify *Bacillus anthracis*, and SNP targets in samples. All the reactions were carried out continuously in thermos cups, and the CatG4R antisense DNA was used as the detection probe of Cas12a reaction. When crRNA bound with the target dsDNA, Cas12a will cleavage CatG4Rz. After CatG4 nucleic acid was added, CatG4 and hemin can form an activated G-quadruplex-hemin complex, which catalyzed ABST^2−^ and H_2_O_2_ to produce ABST^−^ and turn the solution green. If no target, the solution remained colorless.

Pardee et al., 2016 [115] developed an assay to detect SNP between African and American Zika Virus which was named NASBACC. The process of nucleic acid sequence-based amplification (NASBA) began with reverse transcription to create an RNA/DNA duplex. Then, RNase H degraded the RNA to form ssDNA. Using primer containing the T7 promoter, dsDNA was synthesized and then transcribed to generate RNA. In the presence of RNA target and the PAM sequence, the dsDNA was synthesized and cleaved by CRISPR/Cas9. It was unable to activate the sensor H, and the color will not change. In the absence of RNA target, the dsDNA was intact, generating the sensor H trigger sequence, then the sensor H was activated. The activated sensor H regulates translation of LacZ, which regulated color change by converting a yellow substrate (chlorophenol red-b-D-galactopyranoside) to a purple product (chlorophenol red). Blanluet et al., 2022 [116] found that end-point fluorescence was not suitable for distinguishing between WT and SNPs, thus analyzed the Michaelis–Menten kinetic effects of SNP versus WT activation activated Cas12 *trans*-cleavage activity. Through calculating the apparent catalytic efficiency *k ∗ cat/K_M_* to identify SNPs and WT, the authors found that the 60 SNPs yielded a lower *k ∗ cat/K_M_* than the WT.

### 2.3. Comparison of Advantages and Disadvantages of Detection Methods

The detection accuracy and sensitivity of all the above methods are very good. Some of them are quite portable and faster than the traditional detection methods for GMOs, gene-edited products and SNPs. However, those methods still have defects in some aspects, and cannot be well applied in practice.

Wang et al., 2020 [99], Duan et al., 2022 and Wang et al., 2022 [107] use the rapid genomic extraction method. This method is simple, rapid and meet the requirements of on-site detection. However, compared to the genomic extraction kit in the laboratory, the residue of protein, RNA or salt ions may affect the *trans*-cleavage efficiency of Cas effectors. Almost all of the above detection methods require target nucleic acid amplification (Table 2). Isothermal amplification methods do not require precision instruments, and is simple and fast to operate. Compared to isothermal amplification, PCR is time-consuming and not suitable for on-site detection. However, PCR has higher amplification efficiency and accuracy than LAMP, RPA, RAA and RCA methods, and it is widely used for nucleic acid amplification. The fluorescence-based detection requires ultraviolet/blue light sources, fluorescence spectrometer or other instruments that does not require complex data processing and analysis. Some results can be directly judged by the naked-eye through different colors, which is very portable. Although the use of portable instruments, naked-eye detection also requires testing in darker environments. The lateral flow assay-based detection method is highly operable and portable, but it requires the selection of appropriate antigen/antibody, antigen/antibody concentration and buffer, which is more complex than fluorescence-based detection. However, the lateral flow assay-based detection has low throughput, and accuracy dependent on the specificity of the antibody. The electrochemical-based detection method provides linear output, low power consumption, and good resolution, repeatability and accuracy without contamination by other gases. Nevertheless, electrochemical biosensor is affected by the temperature range, cross-influence of different gases and short service life.

Optimization can be performed in three steps—nucleic acid extraction, amplification and readout methods. First, for nucleic acid extraction, especially seed materials, the research and development and innovation of portable simple extraction devices should be sped up. The DNA direct extraction method and a nucleic acid extraction test strip, using cell lysate as template for direct amplification, can omit nucleic acid purification step. However, it is also necessary to consider how to overcome the adverse effects of inhibitors, such as intracellular ions and proteins on nucleic acid amplification. Secondly, for nucleic acid amplification, nucleic acid thermostatic amplification technology, such as RPA, removes the dependence of traditional PCR technology on large-scale instruments and has a good application prospect. However, how to reduce the cost of nucleic acid thermostatic amplification enzyme, improve the stability of transportation and preservation, and optimize the primer design still need to be further studied. Last, the detection is generally divided into real-time detection and end-point detection. The end-point detection, such as a nucleic acid test strip and the chromogenic method, is closer to the fast and visual detection requirements. How to avoid aerosol pollution and ensure sensitivity and specificity are key to application.

## 3. Challenges and Prospects

The CRISPR/Cas detection system exhibits many excellent characteristics, such as low cost, low speed, mild conditions, simple operation, rapid and high accuracy. However, research on the field of the CRISPR/Cas detection system is only into a few years, and still in the laboratory stage, and there are some disadvantages that cannot be ignored. First, the off-target effect is one of the problems since the advent of gene editing, and may lead to false-positive or -negative results, which should be considered [117,118,119,120]. The structure of different Cas effectors and the unsuitable sequence and secondary structure of guide RNA [121,122] have significant influences on the off-target effects. In fact, the mismatch between guide RNA and target is the main reason. Zetsche et al., 2015 [123] found that the mismatch within the first 5 nt on the 5′ end of the spacer sequence can be accurately identified, but the others cannot. Fu et al., 2013 [124] found that the specificity of CRISPR/Cas was complex and depended on the target site. Sometimes, the single and double mismatches in the 3′ end of the guide RNA showed good tolerance, but the double mismatches in the 5′ end shown low activities. At the same time, the author also found that reducing the concentration of the CRISPR/Cas and the guide RNA did not reduce the off-target effects. If the guide RNA has a high GC content, the hybridization between RNA and DNA can be more stable [125]. Constructing high-fidelity Cas9 effectors [126,127], optimizing guide RNA structure [128,129], and high GC content may solve off-target effects. Second, most Cas effectors require a PAM/protospacer-flanking sequence (PFS) contained in the target sequences, in order to accurately identify the target sequence, except the Cas14 effector. Further, different Cas effectors have their own bias for recognizing PAM/PFS (SpCas9, FnCas12a and LbCas12a recognize PAM as NGG, TTN and TTTN). This means that a target sequence may only be recognized by only one Cas effector. Hence, the selection of PAM/PFS limits the use of this method. In some cases, there are mutation sites for which no or no suitable PAM sequence is available, requiring additional insertion. By designing the PAM sequence at the appropriate position of the primer, the amplifications will have the PAM sequence recognized by Cas effectors. Therefore, sequence-independent detection can be performed at any site. By inserting PAM/PFS at suitable locations in the primers, the amplicon with PAM sites can be used for subsequent experiments [106,107,108]. In addition, Cas is a tool for nucleic acid detection and not non-nucleic acid target detection. Nucleic acid aptamers need to be designed to achieve non-nucleic acid target detection by indirectly detecting aptamers. However, PAM has the potential to alter the aptamer concept, thus reducing the binding ability of the aptamer to the target. Third, it is still difficult to achieve standardization, as well as multiple and quantitative detection. The concentration ratio of Cas effectors to RNA, the pH, concentration of Mg^2+^ and Mn^2+^ of the buffer, and the reaction temperature may interfere with the reaction process and sensitivity of Cas effectors. Because of the indiscriminate *trans*-cleavage of Cas effectors, and the easily saturated detection signal, it hinders the multiple and quantitative detection by using CRISPR/Cas detection systems. The standardization problem can be solved to some extent by on-site calibration and unified systems. A droplet-based microfluidic device [130,131] coupled with CRISPR/Cas maybe a good choice for detection. Forth, many methods require nucleic acid amplification before CRISPR/Cas detection to obtain a lower LOD. PCR amplification, RPA or other amplification methods usually suffer from problems such as secondary structures of primers or templates and contaminants, and undoubtedly increase the complexity of detection. At present, there is no better method to avoid this. The only way to minimize the complexity of sample processing is to optimize the amplification methods and procedures. Fifth, all the above methods have different disadvantages and need to be optimized in three steps—nucleic acid extraction, amplification, detection methods, such as low throughput, instrument dependence or complex design. Through combining and optimizing several methods, exploring the optimal detection conditions, simplifying the process of sample pretreatment, reaction steps and readout mode, developing portable devices may be a better choice. Last, the storage and transport of Cas protein and guide RNA are also a challenge for detection. If the storage temperature of Cas protein and guide RNA is not sufficient, repeated freezing and thawing during transportation will cause degradation and inactivation. Although the binding of Cas protein and guide RNA into a binary complex will prevent degradation and inactivation [132], it is not a permanent solution. Lyophilizing Cas protein and guide RNA, or improving the storage and transportation equipment would solve this problem.

The CRISPR/Cas system has unique glamour in high sensitivity and specificity detection, and there is no need for professional experimental steps and analysis. The CRISPR/Cas system can be combined with a variety of amplification methods, readout methods, devices to achieve versatile detection of nucleic acid and non-nucleic acid targets in the fields of clinical diagnosis, environmental testing, food safety, biological breeding and others. While many applications of CRISPR testing have been published, CRISPR nucleic acid testing is still in its infancy and has much room for improvement. With the exploration of CRISPR/Cas systems combined with nanomaterials, 3D printing technology, the internet, big data, automation, and artificial intelligence, it will have a great application prospect in the future.

At present, the biotechnology revolution and industrial transformation are accelerating. As more diverse traits and products continue to emerge, molecular characterization information and related databases are very limited and imperfect, rapid and accurate detection methods will become a great challenge for standard detection, and the optimization of mutation detection technology remains a future endeavor. This should be achieved to accelerate the research into testing standards and methods for biotechnology products, so as to protect the intellectual property rights of researchers and provide strong technical support for national security supervision and monitoring.

## Figures and Tables

**Table 1:** The characteristics of Cas9, Cas12, Cas13 and Cas14.

Cas Protein	Cas9	Cas12a (Cpf1)	Cas12b	Cas13a (C2c2)	Cas14a (Cas12f1)
CRISPR system classification	Class 2 Type II-A	Class 2 Type V-A	Class 2 Type V-B	Class 2 Type VI-A	Class 2 Type V-F1
Nuclease domain	HNH and RuvC	RuvC	RuvC	2 x HEPN	RuvC
PAM/PFS	NGG	(T)TTN	TTN	non-G	no
Guide RNA	sgRNA (~100 nt)	crRNA (40–44 nt)	crRNA (40–44 nt)	crRNA (64–66 nt)	crRNA (~140 nt)
Target	dsDNA	ds/ssDNA	ds/ssDNA	ssRNA	ssDNA
*trans*-cleavage	no	ssDNA	ssDNA	ssRNA	ssDNA

Note: PAM, protospacer-adjacent motif; PFS, protospacer-flanking sequence.

**Table 2:** CRISPR/Cas systems in detection of GMOs, gene-edited products and SNPs.

System Name	Cas Effectors	Target	Amplification	Readout	LOD	Time	References
-	Cas12a	*CaMV35S* promoter	PCR/LAMP	Fluorescence detection/ naked eye	0.05 wt %	≥50 min	[94]
Cas12a-PB	Cas12a	*CaMV35S* promoter/*Lectin* gene	Dual ordinary/rapid PCR/LAMP	Fluorescence detection/ naked eye	0.1 wt %	≥30 min	[95]
MPT-Cas12a/13a	Cas12a/Cas13a	*CaMV35S* and T-*nos*	Multiplex PCR	Fluorescence detection/ naked eye	13 copies/11 copies	<2 h	[96]
PE-MC/SDA-CRISPR/Cpf1	Cpf1	*CaMV35S*	Multiple cascade strand displacement amplification	Fluorescence detection/ naked eye	14.4 fM	~3 h	[97]
RPA-Cas12a-FS	Cas12a	Foodborne pathogenic bacteria/GMO/meat adulteration	RPA	Fluorescence detection	10 copies (GMOs)	~45 min	[98]
RPA-Cas12a-LFB	Cas12a	P-*CaMV*35S/T-*nos*	RPA	Fluorescence detection/ lateral flow strip	10 copies/0.01 wt %	~40 min	[99]
-	Cas12a/Cas13a	Transgenic rice/ASFV/miRNAs	PCR/RPA	Naked eye	0.01 wt % (GMOs)	~1 h	[87]
CASLFA	Cas9	Pathogenic microorganism/GMO/virus	PCR/RPA	Fluorescence detection/ lateral flow strip	0.01 wt % (GMOs)	~40 min	[100]
-	Cas12a	P-*CaMV*35S and *HPT/NPTII* and T-*nos*	LAMP	Fluorescence detection/ naked eye/lateral flow strip	25 copies/100 copies	≤40 min	[101]
-	Cas12a	T-*nos*	PCR/RAA	Fluorescence detection/ naked eye	0.1 wt %/0.1 to 40 wt % semi-quantified	>1 h	[102]
-	Cas12a	GM soybean	Amplification-free	Fluorescence detection/ electrochemistry	0.3 fM	~1 h	[103]
isoCRISPR	Cas12a	GMO	RCA	Fluorescence detection/ electrochemistry	45 pM	~2.5 h	[104]
PCR/RNP	Cas9/Cpf1	Gene-edited wheat/rice	PCR	Gel analysis	WT: D1/D1: WT of 1:20	>3 h	[105]
-	Cas12a	Gene-edited Thp-1 cells	PCR	Fluorescence detection	10 pg	~2 h	[106]
Cas12aFVD	Cas12a	Gene-edited rice	PCR/RPA	Fluorescence detection/ naked eye	12 copies	≤40 min	[107]
HOLMESv2	Cas12b	SNP/SNM/RNA	Asymmetric PCR/LAMP	Fluorescence detection	10^−8^ nM	<2.5 h	[108]
HOMLES	Cas12a	SNP	PCR	Fluorescence detection	10 aM	~1 h	[109]
CDetection	Cas12b	SNP	PCR/RPA	Fluorescence detection	10^−18^ M	~1 h	[110]
SHERLOCK	Cas13a	Pathogenic bacteria/ SNPs of ZIKV	RPA/RT-RPA	Fluorescence detection	0.1% of background DNA (SNPs)	~1 h	[71]
Cas14a-DETECTR	Cas14a	SNP	Phosphorothioate amplification approach	Fluorescence detection	-	~1 h	[73]
MeCas12a	Cas12a	SNP	RAA/RT-RAA	Fluorescence detection/ naked eye	5 copies	~45 min	[111]
-	Cas12a	SNP	PCR	Fluorescence detection/ naked eye	-	~1.5 h	[112]
-	Cas12a	SNV	PCR	Fluorescence detection/ electrochemistry	10 aM	>1 h	[113]
-	Cas12a	*Bacillus anthracis/*SNP	PCR/RPA	Fluorescence detection/ naked eye	1 copy	~1.5 h	[114]
NASBACC	Cas9	SNP between African and American ZIKV	Nucleic acid sequence-based amplification	Naked eye	2.8 fM	~3 h	[115]
-	Cas12a	SNP	-	Michaelis–Menten kinetic effects	-	-	[116]

Note: GMO, genetically modified organism; SNP, single-nucleotide polymorphism; SNM, single-nucleotide mismatch; SNV, single-nucleotide variant; PCR, polymerase chain reaction; LAMP, loop-mediated isothermal amplification; RPA, recombinase polymerase amplification; RAA, recombinase-aided amplification; RCA, rolling circle amplification; RT-RPA, reverse transcription-RPA; RT-RAA, reverse transcription-RAA.

## Data Availability

Data is contained within the article.
